# Changes in Glycemic Control and Lipid Profiles Following COVID-19 Infection in Adults with Type 2 Diabetes: A Retrospective Study in Saudi Arabia

**DOI:** 10.3390/pathogens15090934

**Published:** 2026-09-04

**Authors:** Yazeed Alshuweishi, Jehad A. Aldali, Hamood Alsudais, Abdulrahman Alshalani, Abdulaziz M. Almuqrin, Badi A. Alotaibi

**Affiliations:** 1 Chair of Medical and Molecular Genetics Research, Department of Clinical Laboratory Sciences, College of Applied Medical Sciences, King Saud University, Riyadh 12372, Saudi Arabiahalsudais@ksu.edu.sa (H.A.); aalmuqrin@ksu.edu.sa (A.M.A.); 2 Department of Pathology, College of Medicine, Imam Mohammad Ibn Saud Islamic University (IMSIU), Riyadh 13317, Saudi Arabia; 3 Department of Clinical Laboratory Sciences, College of Applied Medical Sciences, King Saud Bin Abdulaziz University for Health Sciences, Ministry of National Guard Health Affairs, Riyadh 11481, Saudi Arabia; 4 King Abdullah International Medical Research Centre, Riyadh 11481, Saudi Arabia

**Keywords:** COVID-19, dyslipidemia, glycemia, type 2 diabetes, pre-COVID, post-COVID

## Abstract

**Background:** The clinical course and mortality risk of COVID-19 in patients with type 2 diabetes (T2D) remain elevated. Understanding its pathogenesis is essential for prognostic and therapeutic strategies. Viral infections can disrupt host glucose and lipid metabolism. This study evaluated changes in glycemic and lipid parameters following COVID-19 infection in T2D patients. **Methods:** A retrospective analysis was conducted on T2D patients who tested positive for COVID-19 between April 2020 and December 2021. Laboratory results before and after infection were compared to assess changes in glycemic and lipid markers. **Results:** The study included 969 patients with T2D; 56.7% were female, and the median age was 58 years (IQR: 49–67). Hypertension, asthma, and chronic kidney disease were present in 51.5%, 14.2%, and 7.2% of patients, respectively. Fasting blood glucose did not change significantly from before to after COVID-19 infection [7.9 mmol/L (IQR: 6.2–10.6) vs. 7.8 mmol/L (IQR: 6.2–10.2)], nor did HbA1c [7.6% (IQR: 6.7–9.1) vs. 7.6% (IQR: 6.5–9.2)]. In contrast, modest but statistically significant reductions were observed in triglycerides [1.47 mmol/L (IQR: 1.13–2.00) vs. 1.44 mmol/L (IQR: 1.08–1.94)], total cholesterol [4.3 mmol/L (IQR: 3.67–5.03) vs. 4.1 mmol/L (IQR: 3.55–4.93)], and LDL cholesterol [2.7 mmol/L (IQR: 2.11–3.45) vs. 2.5 mmol/L (IQR: 1.98–3.23)]. HDL cholesterol remained unchanged [1.0 mmol/L (IQR: 0.90–1.21) vs. 1.0 mmol/L (IQR: 0.89–1.22)]. **Conclusions:** COVID-19 infection was not associated with significant long-term changes in glycemic control among patients with T2D, as fasting blood glucose and HbA1c levels remained broadly stable after infection. In contrast, significant reductions were observed in total cholesterol, LDL cholesterol, and triglyceride levels, while HDL cholesterol remained unchanged. These findings indicate that changes following COVID-19 infection were more apparent in lipid parameters than in glycemic markers.

## 1. Introduction

The global outbreak of COVID-19, caused by SARS-CoV-2 infection, has not only posed an unprecedented threat to public health but also promoted a significant number of investigations into its multifaceted impacts on various physiological systems [1]. One such area of extensive study is the potential effects of COVID-19 on lipid and glycemic markers. This subject has gained considerable attention due to its potential implications for individuals with metabolic disorders such as type 2 diabetes (T2D) and those at risk of developing metabolic disorders [2]. SARS-CoV-2 primarily targets cells expressing the angiotensin-converting enzyme 2 (ACE-2) receptors since ACE-2 is the main receptor for virus attachment and entry [3]. ACE2 is widely expressed not only in the respiratory tract but also in metabolic tissues such as adipose tissue, the pancreas, liver, and intestines [4]. Therefore, it is not unexpected that SARS-CoV-2 infection may lead to disturbances in metabolic function [5].

Viral infections have been demonstrated to disrupt metabolic homeostasis, leading to significant alterations in glycemic markers and lipid profiles. For instance, infection with the hepatitis C virus (HCV) has been associated with insulin resistance and the development of T2D [6]. Similarly, HIV infection and its treatment regimens have been linked to dyslipidemia, characterized by elevated triglycerides and decreased HDL cholesterol levels [7].

These metabolic disturbances are thought to arise from a combination of chronic systemic inflammation, direct viral effects on insulin signaling pathways, and antiviral therapy-induced metabolic derangements. As such, viral infections may increase the risk of metabolic syndrome and cardiovascular disease, underscoring the importance of metabolic monitoring in infected individuals. More recently, the emergence of the COVID-19 pandemic has led to a growing number of studies investigating the virus’s profound effects on metabolic pathways, with evidence suggesting that SARS-CoV-2 infection can induce or exacerbate disturbances in glucose and lipid metabolism, further highlighting the interplay between viral infections and metabolic health [8]. In addition to actively participating in vital cellular processes, including molecular trafficking, lipids are structural elements of the host’s cellular membranes and play key roles during viral attachment, internalization, packaging, and release [9]. Along with glucose, lipids are also crucial for energy supply and intracellular signaling, and it has been shown that the infectivity of SARS-CoV-2 is mediated by cholesterol content in membrane lipid rafts in animal models [9].

T2D is a known risk factor for the onset of infections, and people with T2D are often older, have other comorbidities, and exhibit significant metabolic, pathophysiologic, and immunologic alterations, which collectively increase susceptibility to COVID-19. In Saudi Arabia, the earliest national data showed that the second most common comorbidities among 1519 confirmed cases of COVID-19 infection were T2D patients [10]. This might be due to its linked metabolic complications, and that hyperglycemia predicts severity in previous SARS-CoV-1 and Middle East Respiratory Syndrome coronavirus-CoV infections [11]. Notably, a higher frequency of hyperglycemia in COVID-19-positive patients was observed regardless of whether a patient had diabetes mellitus before the infection [12]. In a study conducted in Italy on 271 patients who had been admitted with COVID-19, hyperglycemia was found to be independently linked to a higher death rate [13]. Additionally, SARS-CoV-2 infections alter their host cells’ lipid profiles and metabolism, like the case with other viral infections such as MERS and SARS-CoV-2002 [14].

Following the acute stage of the SARS-CoV-2 disease course, further research is needed to determine the long-term effects of COVID-19 infection on glucose and lipid metabolism. Although many studies on the relationship between diabetes and COVID-19 used clinical measures of disease severity and outcomes in COVID-19 patients, less is known about the long-term impact of COVID-19 infection on individual parameters of lipid or glucose metabolism in patients with chronic diseases such as T2D. Hence, this retrospective analysis sought to examine changes in metabolic markers following COVID-19 infection among Saudi T2D patients. We hypothesized that since SARS-CoV-2 enters host cells mainly through ACE-2 receptors [3], which are expressed in metabolic tissues, including in adipose tissue, the pancreas, liver, and intestines [4], COVID-19 infection may disrupt metabolic homeostasis and result in measurable alterations in glycemic and lipid markers among patients with T2D. Here, the medical records of each patient included in the study were retrieved, and lipid profiles and glycemic markers measured at least one month before and post-COVID-19 infection were compared to allow direct analysis. The aim of this study was to evaluate the impact of COVID-19 infection on glycemic control and lipid profiles in adults with type 2 diabetes by comparing fasting blood glucose, HbA1c, triglyceride, total cholesterol, LDL, and HDL levels before and after confirmed COVID-19 infection.

## 2. Materials and Methods

### 2.1. Study Design

The current retrospective cohort study utilizes data from the King Abdullah International Medical Research Centre (KAIMRC), Ministry of National Guard, Health Affairs, Riyadh, Saudi Arabia. The ethical approval for this study was obtained from the Institutional Review Board of KAIMRC on 15 August 2023 (IRB:2116/23 and study number NRC23R/457/08). In this study, data on adult patients with type T2D who were diagnosed with COVID-19 between April 2020 and December 2021 using fast antigen testing or polymerase chain reaction testing were obtained from the Best Care electronic health record system. Exclusion criteria included individuals who were previously diagnosed with type 1 diabetes. Two assessment timepoints were defined: the pre-COVID-19 timepoint, comprising the closest available laboratory measurement obtained 1–12 months before confirmed COVID-19 infection, and the post-COVID-19 timepoint, comprising the closest available measurement obtained 1–12 months after infection. For each patient, biochemical measurements were obtained from the same selected pre- and post-COVID-19 laboratory encounters. The intervals between the pre-COVID-19 assessment and COVID-19 diagnosis and between diagnosis and the post-COVID-19 assessment were calculated in days. The distributions of these intervals were summarized using the median, interquartile range, and range. Paired within-patient comparisons were performed, with each patient’s pre-COVID-19 value serving as the comparator for the corresponding post-COVID-19 value. This approach was used to minimize the influence of inter-individual variability and baseline treatment differences, as the comparison was performed within the same patient rather than between separate patient groups. To minimize the potential influence of time-dependent confounding factors, the laboratory results closest to the date of COVID-19 diagnosis were preferentially selected within each predefined window, while maintaining a minimum interval of one month before and after infection. Therefore, the follow-up period for laboratory comparison was limited to a maximum of 12 months after COVID-19 infection.

Dedicated research team members contacted the medical record unit, research department, and data management section at KAIMRC for data collection. Baseline characteristics of all patients include sex, age, hypertension, coronary artery disease, chronic obstructive pulmonary disease (COPD), asthma, end-stage renal disease (ESRD), chronic kidney disease (CKD), osteoporosis, rheumatoid arthritis, and lupus erythematosus. Laboratory data of glycemic markers includes fasting blood glucose (FBG) and hemoglobin A1c (HbA1c) levels. In addition, data on lipid profiles were collected, which include triglycerides (TG), total cholesterol (TC), low-density lipoprotein (LDL), and high-density lipoprotein (HDL) concentrations. Missing values were not imputed. Analyses were conducted using complete paired pre- and post-COVID-19 measurements for each outcome. Consequently, the analytical sample size differed across FBG, HbA1c, TG, TC, LDL-C, and HDL-C. Outcome-specific sample sizes and characteristics are reported in Supplementary Table S1.

### 2.2. Statistical Analysis

Statistical analyses were performed using SPSS software version 25.00 (IBM Corp., Armonk, NY, USA), and graphical representations were generated using GraphPad Prism version 9.4.1 (GraphPad Software Inc., San Diego, CA, USA). Descriptive data were generated for all baseline characteristics and were summarized using medians and interquartile ranges (IQRs) for continuous data or percentages and frequencies for categorical data. The normality of the data was determined with the Shapiro-Wilk test. The distributions of the metabolic variables were assessed using the Shapiro-Wilk test. Because the variables were non-normally distributed, the results are presented as medians and interquartile ranges. Paired pre- and post-COVID-19 measurements were compared using the Wilcoxon matched-pairs signed-rank test. To describe the magnitude and precision of lipid changes, the Hodges–Lehmann estimate of the paired difference and its 95% confidence interval were calculated. Matched-pairs rank-biserial correlation was reported as a standardized effect size. Paired changes were defined as the post-COVID-19 concentration minus the pre-COVID-19 concentration; therefore, negative estimates indicated reductions. A *p*-value of less than 0.05 was considered significant.

If the paired comparison demonstrated a statistically significant difference between the pre- and post-COVID-19 measurements for a glycemic or lipid parameter, exploratory regression analyses were subsequently performed to examine whether selected baseline characteristics were associated with that outcome. Candidate predictors were first evaluated in univariable analyses using Pearson’s or Spearman’s correlation, as appropriate. To minimize premature exclusion of potentially relevant covariates at this exploratory screening stage, a deliberately liberal inclusion threshold of *p* < 0.50 was used. This threshold was used solely for candidate-variable screening and was not interpreted as evidence of statistical significance. The rationale for using a permissive screening criterion was based on the broader purposeful-selection principle that conventional thresholds such as *p* < 0.05 may exclude variables that could become relevant after adjustment for other covariates [15,16]. Variables meeting the screening criterion were subsequently entered into the multivariable regression models. Statistical significance in the final multivariable models was defined as *p* < 0.05. Because the analyses were exploratory and some comorbidities were infrequent, regression coefficients for rare conditions were interpreted cautiously and primarily as hypothesis-generating rather than confirmatory.

## 3. Results

### 3.1. Study Population

**Figure 1:**
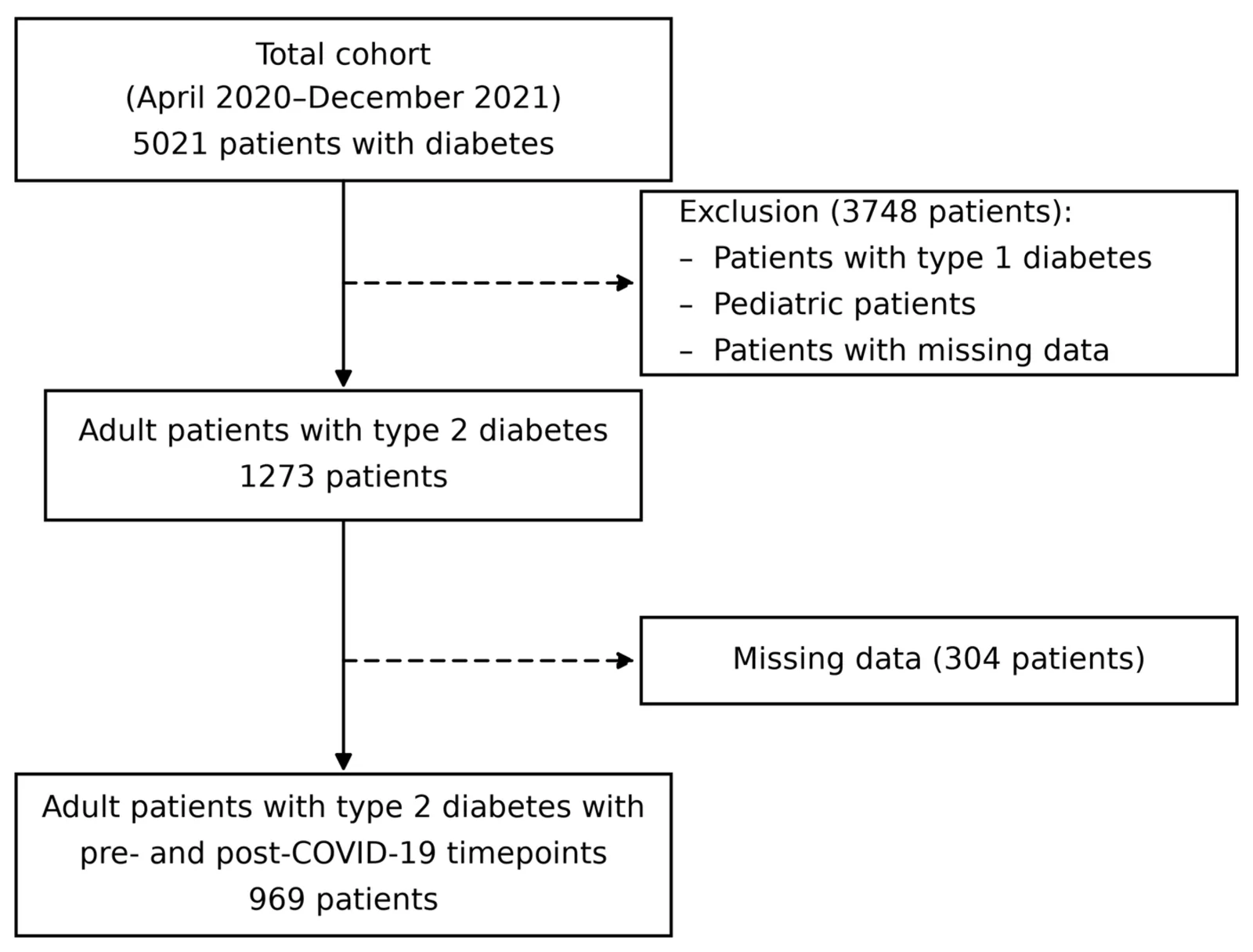
Flowchart of the study design. This flowchart illustrates the study design used to compare lipid and glycemic parameters before and after COVID-19 infection in patients with T2D.

The flowchart is illustrated in Figure 1. Out of 5021 patients with diabetes identified between April 2020 and December 2021, a total of 3748 were excluded due to having type 1 diabetes or being pediatric cases. This yielded 1273 adult T2D patients. An additional 304 patients were excluded due to missing data, resulting in a final sample of 969 T2D patients. Table 1 presents the summary of the demographic and clinical features of the 969 type 2 diabetes participants in the trial after COVID-19 infection. The median age of the study subjects was 58 (IQR: 49–67), with approximately 56.7% being female, and 43.3% being male. For each patient, biochemical measurements were obtained from the same selected pre- and post-COVID-19 laboratory encounters. The median interval between the pre-COVID-19 assessment and COVID-19 diagnosis was 116.0 days (IQR: 70.3–180.8; range: 30–365 days), while the median interval between diagnosis and the post-COVID-19 assessment was 119.5 days (IQR: 74.3–192.8; range: 30–364 days). Regarding comorbidities, 51.5% of the participants had hypertension, 13.2% had asthma, and 7.2% had chronic kidney disease.

Table 2 presents the medications used by patients throughout the study period, including before, during, and after COVID-19 infection, to provide context for interpreting changes in metabolic profiles. To characterize changes in glycemic and lipid parameters following COVID-19 infection, this study compared the levels of individual glycemic and lipid markers measured before and after infection.

### 3.2. Changes in Glycemic Markers Before and After COVID-19 Infection

**Figure 2:**
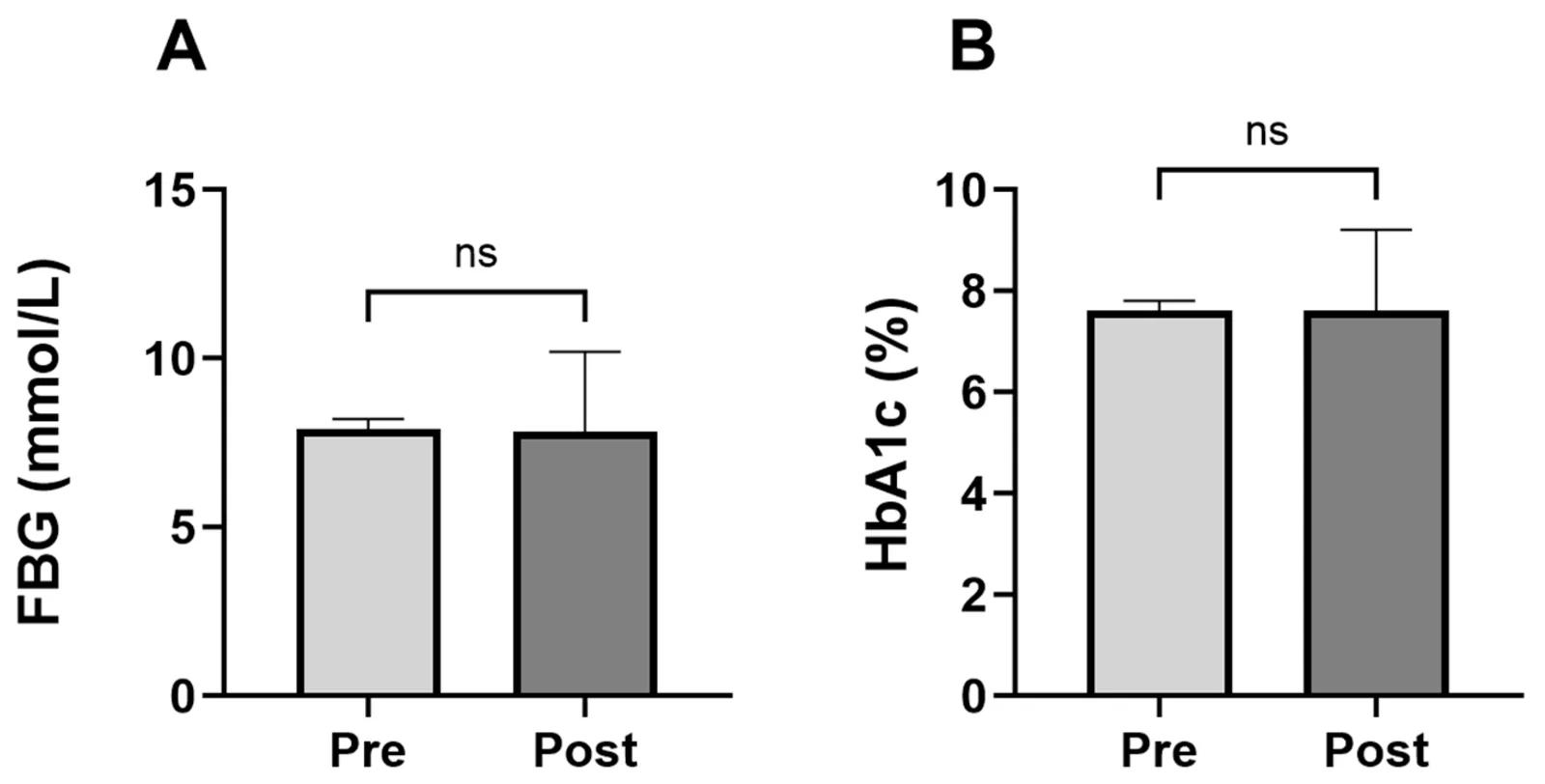
Pre- and post-COVID-19 glycemic control. (**A**) Pre- and post-timepoint mmol/L fasting blood glucose (FBG). (**B**) Pre- and post-timepoint HbA1c percentages. Data are reported as median and interquartile range (IQR). ns, not significant (*p* > 0.05).

Figure 2 shows the fasting blood glucose (FBG) and HbA1c levels examined pre- and post-COVID-19 infection. When laboratory tests were compared, as shown in Figure 2A, the median of FBG from T2D patients pre-COVID-19 infection was 7.9 (IQR: 6.2–10.6), and this was not statistically different from their levels reported post-COVID infection (7.8; IQR: 6.2–10.2). Similarly, no substantial differences were observed in the levels of HbA1c among T2D patients measured pre- and post-COVID-19 infection (Figure 2B; 7.6 (IQR: 6.7–9.1) vs. 7.6 (IQR: 6.5–9.2)).

### 3.3. Changes in Lipid Parameters Before and After COVID-19 Infection

**Figure 3:**
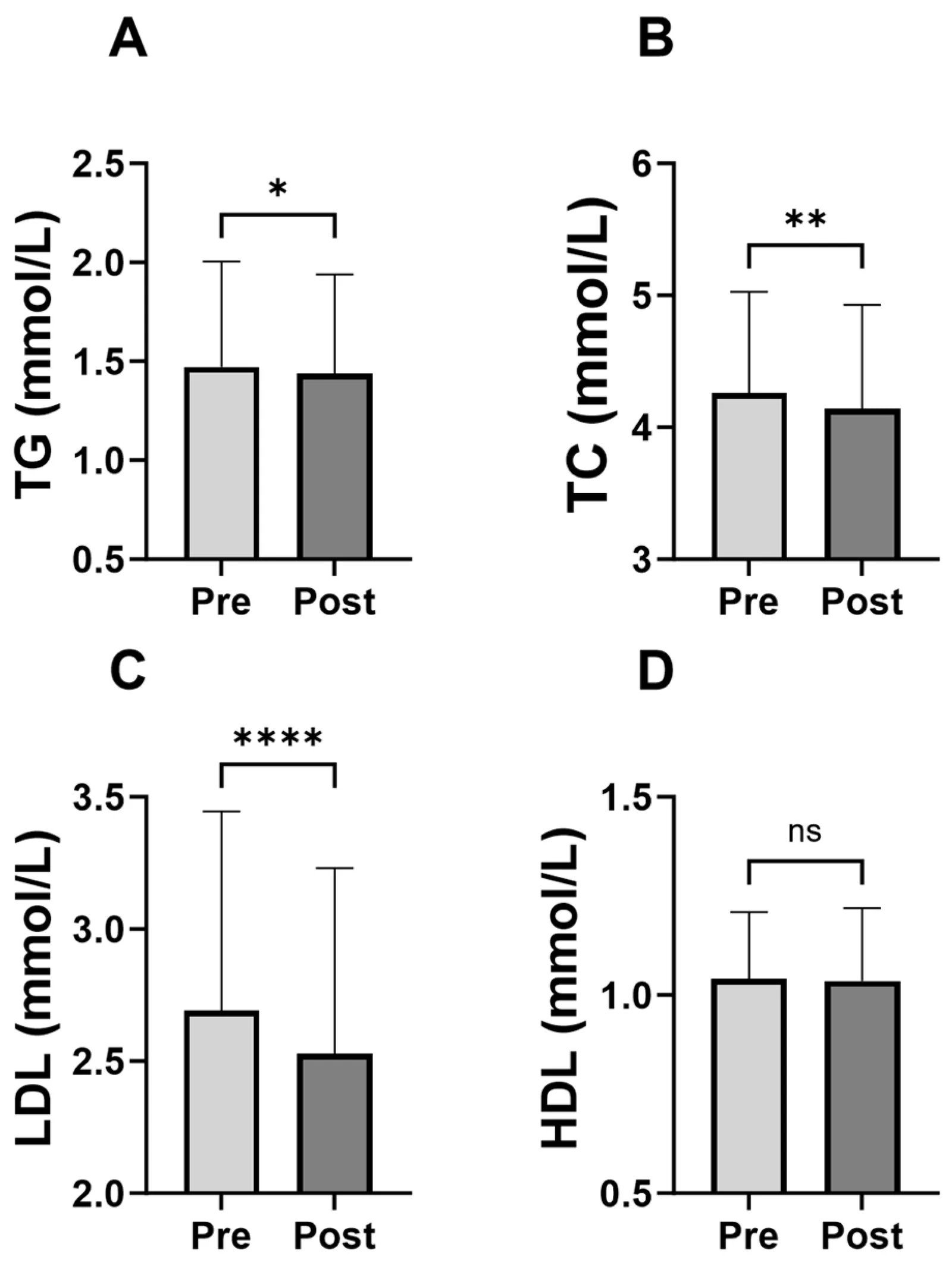
Lipid profile measurements pre- and post-COVID-19 infection in patients with T2D. (**A**) Triglyceride (TG) concentration, (**B**) total cholesterol (TC) concentration, (**C**) low-density lipoprotein (LDL) concentration, and (**D**) high-density lipoprotein (HDL) concentration before and after COVID-19 infection. Data are reported as median and interquartile range (IQR). Statistical significance is indicated as follows: * *p* < 0.05; ** *p* < 0.01; **** *p* < 0.0001. ns, not significant.

Figure 3 presents the changes in lipid values measured among study patients pre- and post-COVID-19 infection. As demonstrated in Figure 3A, there was a modest yet significant reduction in the level of triglycerides (TG) post-COVID-19 infection when compared to the TG level measured pre-COVID-19 infection (1.47 (IQR: 1.13–2.00) vs. 1.44 (IQR: 1.08–1.94)). Moreover, statistically significant reductions were observed after COVID-19 infection in the level of TC (Figure 3B; 4.3 (IQR (3.67–5.03)) vs. 4.1 (IQR: 3.55–4.93)) and LDL (Figure 3C; 2.7 (IQR: 2.11–3.45) vs. 2.5 (IQR: 1.98–3.23)), when compared to their levels measured before the infection. No changes were seen in the level of HDL reported pre- and post-COVID-19 infection (Figure 3B; 1.0 (IQR: 0.90–1.21) vs. 1.0 (IQR: 089–1.22)).

To evaluate the magnitude of the paired lipid changes, median patient-level changes and matched-pairs rank-biserial correlations were calculated (Table 3). TG showed a median change of −0.030 mmol/L (IQR: −0.350 to 0.235), with a negligible effect size (r_rb_ = −0.090; *p* = 0.0422). TC showed a median change of −0.040 mmol/L (IQR: −0.488 to 0.348), with a small effect size (r_rb_ = −0.112; *p* = 0.0084). LDL-C showed the largest reduction, although the effect remained small, with a median change of −0.120 mmol/L (IQR: −0.520 to 0.285; r_rb_ = −0.217; *p* < 0.0001). HDL-C remained unchanged, with a median change of 0.000 mmol/L (IQR: −0.090 to 0.090) and a negligible effect size (r_rb_ = −0.021; *p* = 0.6319). Overall, the statistically significant reductions in TG, TC, and LDL-C were small in magnitude.

### 3.4. Regression Model for Tg, Tc, and Ldl

Table 4 shows the analysis of the correlation between TG, TC, and LDL with baseline characteristic variables, including sex, age, and comorbidities in T2D. The correlations between TG and sex, COPD, ESRD, CKD, and lupus erythematosus resulted in a *p*-value of less than 0.5. Variables meeting the prespecified exploratory screening criterion of *p* < 0.50 (coronary artery disease, COPD, ESRD, CKD, diabetes, and arthritis) were entered into the corresponding multivariable regression model. Furthermore, sex, hypertension, coronary artery disease, asthma, ESRD, CKD, osteoporosis, and rheumatoid arthritis were included in the LDL regression model.

Table 5 presents the multivariable regression models examining factors associated with triglyceride (TG), total cholesterol (TC), and low-density lipoprotein cholesterol (LDL-C) levels. Candidate predictors identified during the univariable screening were entered into the respective models. In the TG model, sex (standardized β = 0.095, *p* < 0.01) and the interval between COVID-19 diagnosis and post-COVID-19 testing (standardized β = 0.365), *p* < 0.001) were positively associated with TG levels, whereas lupus erythematosus was negatively associated with TG (standardized β = −0.123, *p* < 0.001). However, this finding should be interpreted cautiously because lupus erythematosus was present in only four patients, resulting in a potentially unstable estimate. The TG model explained 15.9% of the variance in TG levels (R^2^ = 0.159). None of the included predictors were significantly associated with TC or LDL-C, and these models explained only 0.7% and 1.6% of the variance, respectively.

In sensitivity analyses stratified by the post-COVID-19 testing interval, the median within-patient TG changes were −0.07 mmol/L (IQR: −0.36 to 0.22), −0.02 mmol/L (IQR: −0.29 to 0.21), and −0.02 mmol/L (IQR: −0.35 to 0.32) in the 1–3-, >3–6-, and >6–12-month groups, respectively. The magnitude of TG change did not differ significantly across these groups (Kruskal–Wallis *H* = 0.867, *p* = 0.648). Similarly, no significant difference in TG change was observed across the pre-COVID-19 interval categories (*H* = 1.869, *p* = 0.393; Supplementary Table S3).

## 4. Discussion

COVID-19, first detected in Wuhan, China, in December 2019, led to a global pandemic that had caused more than 6.5 million reported deaths worldwide by late 2022 [17]. It was initially assumed to be a pathogen that affects the respiratory system primarily; its impacts on numerous organ systems are being gradually recognized, with several studies analyzing its implication in diverse disease states. COVID-19 infection has been shown to increase mortality and severity of T2D states [18]. Consistently, Khamidullina et al. reported that hospitalized COVID-19 patients with T2D had higher in-hospital mortality and worsened cardiovascular outcomes during follow-up compared with those without T2D [18].

Lipid metabolism plays a critical role in various stages of the viral life cycle, including membrane integrity, viral replication, endocytosis, and exocytosis [19]. Evidence from previous outbreaks, such as SARS-CoV-1, has demonstrated persistent alterations in lipid metabolism following recovery, indicating a potential biological link between viral infection and lipid dysregulation [20]. According to clinical results, patients with acute Epstein–Barr Virus (EBV) infection had reduced HDL, TC, and LDL levels in comparison to control subjects [21].

Similarly, patients with hepatitis B had lower levels of LDL and HDL [22], while patients with HIV exhibited higher TG and lower TC and HDL compared to controls [23]. In a similar vein, research found cytomegalovirus (CMV) infection was associated with higher HDL and lower TG in females with extreme obesity but lower HDL in healthy weight females [24]. Large-scale studies, such as the International Monitoring Dialysis Outcomes (MONDO) study, have also highlighted an inverse association between lipid levels and both all-cause and infection-related mortality [25]. Furthermore, another study found that recovered SARS patients exhibited differences in lipid metabolism [20].

The objective of this investigation was to compare the levels of fasting blood glucose, HbA1c, triglycerides, total cholesterol, LDL, and HDL in adults with T2D before and after a confirmed COVID-19 infection in order to assess the impact of the infection on glycemic control and lipid profiles.

The present study examined the demographic and clinical characteristics of patients with T2D following COVID-19 infection and evaluated changes in their glycemic and lipid profiles. The study population consisted of a slightly higher proportion of females than males. Hypertension was the most common comorbidity, while asthma and chronic kidney disease were also present among a smaller proportion of participants. These comorbidities are clinically relevant because they may independently influence metabolic control and cardiovascular risk in individuals with T2D diabetes.

The medications used before, during, and after COVID-19 infection were also considered to provide an appropriate clinical context for interpreting changes in metabolic parameters. Treatment modifications following infection, particularly changes in glucose-lowering and lipid-lowering therapies, may influence laboratory measurements independently of the direct effects of COVID-19. Therefore, observed changes should be interpreted in relation to patients’ ongoing clinical management and medication use.

The findings showed that fasting blood glucose and glycated hemoglobin levels remained broadly unchanged following COVID-19 infection. This indicates that no sustained deterioration in glycemic control was observed during the post-COVID-19 follow-up period examined. The absence of a persistent change in glycemic markers may also reflect continued diabetes monitoring, medication adherence, and adjustments to glucose-lowering treatment following infection.

These results align with those presented by Wong et al. [26] who observed no statistically significant differences in post-index HbA1c levels or weight among patients with T2D who had confirmed COVID-19 infection when compared to matched controls over a one-year follow-up period. In a similar vein, Laurenzi et al. [27] indicated that dysglycemia observed at the point of hospital admission in COVID-19 patients predominantly improved during the recovery phase, with HbA1c levels showing no significant differences among normoglycemic, hyperglycemic, and diabetes groups throughout the observation period. These studies provide context for the present observation that no sustained deterioration in glycemic markers was detected during the available follow-up period in this cohort. However, because information on COVID-19 severity, hospitalization, oxygen or respiratory support, vaccination status, and viral variant was unavailable, this observation should not be generalized to all patients with T2D following COVID-19 infection.

However, various studies have reported conflicting results. Starling et al. [28] demonstrated through continuous glucose monitoring that glycemic abnormalities can persist for a minimum of two months following recovery from COVID-19, with patients presenting with new-onset hyperglycemia at admission continuing to exhibit dysglycemia at the six-month follow-up. Similarly, Ohkuma et al. [29] found that in a Japanese cohort of T2D patients, the annual mean levels of HbA1c, total cholesterol, and triglycerides were all significantly elevated during the COVID-19 pandemic compared to the pre-pandemic period. These inconsistencies may be attributed to variations in the severity of COVID-19, the duration of follow-up, the characteristics of the background population, and the level of diabetes management throughout the study period, underscoring that the impact of COVID-19 on glycemic control may be contingent upon context and severity rather than being uniform.

In contrast to the stable glycemic findings, modest reductions were observed in the levels of triglycerides, total cholesterol, and low-density lipoprotein cholesterol after COVID-19 infection. High-density lipoprotein cholesterol remained relatively unchanged. Effect estimates and confidence intervals indicated that the lipid reductions were modest. The TG finding was borderline and had a negligible effect size, whereas TC showed a small reduction. LDL-C demonstrated the largest and most consistent reduction, although its effect size remained small. These changes may have limited clinical relevance at the individual-patient level; however, even modest lipid reductions could potentially be meaningful if sustained over time or considered at the population level. These changes may reflect intensified lipid management, improved adherence to treatment, dietary modifications, weight changes, or increased healthcare contact following COVID-19 infection. They may also be influenced by the timing of laboratory testing and the duration between infection and follow-up measurement. Although triglycerides, total cholesterol, and LDL-C decreased significantly following COVID-19 infection, the absolute and percentage changes were modest, with negligible-to-small effect sizes. Therefore, the statistical significance of these findings should not be interpreted as evidence of clinically meaningful improvement in lipid profiles. HDL-C remained essentially unchanged, further indicating that COVID-19 infection had limited overall influence on lipid parameters in this cohort. These findings should consequently be interpreted cautiously, as the observed statistical differences may reflect the study’s sample size and within-patient variability rather than changes of substantial clinical importance.

The direction of these lipid alterations is consistent with other earlier studies. Almas et al. [30] discovered that LDL-C, HDL-C, triglycerides, and total cholesterol were all significantly lower in COVID-19 patients than in uninfected controls, with the magnitude of the reduction increasing with disease severity, broadly supporting the pattern of reduced TG, TC, and LDL observed in the current cohort. Similarly, Li et al. [31] found that LDL-C and HDL-C levels, which were reduced during acute infection, showed a time-dependent, partial normalization three to six months after discharge, particularly among patients with more severe disease, implying that COVID-19 lipid alterations follow a dynamic trajectory during the recovery period.

Conversely, other studies have indicated a contrasting trend. Xu et al. [32], utilizing a substantial cohort of participants from the United States Veterans Affairs, showed that the post-acute phase of COVID-19 was linked to a markedly heightened risk of developing dyslipidemia, characterized by elevated total cholesterol, increased triglycerides, high LDL-C, and decreased HDL-C, with the risk escalating in relation to the severity of the infection. Similarly, López-Hernández et al. [33] illustrated that dysregulation of lipid pathways, which include sterols, bile acids, phospholipids, and sphingolipids, may endure for as long as two years following recovery from COVID-19, exhibiting a complex pattern of both increased and decreased lipid markers. These conflicting results imply that the nature and duration of lipid change post-COVID-19 could be influenced by variables such as the severity of the infection, the elapsed time since infection, pre-existing metabolic conditions, and alterations in concurrent medications, including lipid-lowering treatments, rather than indicating a singular, uniform biological reaction.

The reduction in low-density lipoprotein cholesterol is potentially relevant because patients with T2D are already at increased risk of cardiovascular disease. However, the observational nature of the study prevents the observed lipid changes from being attributed directly to COVID-19 infection. Accounting for factors relating to the initiation, discontinuation, or dose adjustment of lipid-lowering medications is therefore essential when interpreting these findings. Other factors, including illness severity, hospitalization, changes in physical activity, nutritional status, and post-infection weight loss, may also have contributed to the observed changes.

Several demographic and clinical characteristics were initially examined for their relationships with triglyceride, total cholesterol, and low-density lipoprotein cholesterol levels. Variables showing evidence of correlations were subsequently included in the multivariable regression models. Although several comorbidities met the criteria for inclusion, most were not independently associated with total cholesterol or low-density lipoprotein cholesterol after adjustment. This indicates that the variables included in the models explained only a limited proportion of the variation in these lipid outcomes.

A positive association was identified between triglyceride levels and sex, suggesting that triglyceride responses may differ between males and females. The interval between COVID-19 infection and post-infection laboratory testing was also positively associated with triglyceride levels. This finding suggests that the timing of follow-up assessment may affect the detection and interpretation of post-COVID-19 metabolic changes. However, sensitivity analyses showed that the magnitude of within-patient triglyceride change did not differ significantly across the 1–3-, >3–6-, and >6–12-month testing windows. These analyses address related but distinct questions: the regression model evaluated the association between the exact testing interval and the absolute triglyceride concentration, whereas the stratified analysis assessed whether the change from the pre-COVID-19 value differed across broader timing categories. Moreover, categorizing a continuous interval reduces temporal resolution and statistical power. Collectively, the findings provide no clear evidence that measurement timing modified the pre-to-post triglyceride change, although an influence of temporal heterogeneity on absolute triglyceride levels cannot be excluded.

The identified sex-based disparity in triglyceride levels aligns with the current literature that outlines differences in sex-specific lipid profiles. Ramírez-Marín et al. [34] observed that women typically present with elevated HDL-C and reduced LDL-C compared to men, a trend significantly shaped by endogenous sex hormones. They also emphasized the sex-specific variations in lipid and pharmacological responses during SARS-CoV-2 infection, which supports a biological foundation for the observed sex-related differences in triglyceride levels in the current regression analysis. Previous population-based research by Kolovou et al. [35] similarly indicated that while women generally have higher total cholesterol and HDL-C levels than men, the association between triglycerides and sex may differ across various subgroups, highlighting the intricate nature of sex-based lipid disparities.

This study’s findings reveal a positive correlation between triglyceride levels and the duration between infection and post-COVID-19 testing, which somewhat contrasts with the time-dependent partial normalization of lipid parameters documented by Li et al. [31]. However, it is consistent with the evidence presented by López-Hernández et al. [33], indicating that dysregulation of lipid pathways may either persist or evolve over prolonged periods following recovery from COVID-19. Collectively, these results imply that the timing of lipid evaluations in relation to infection could be a significant, albeit inconsistently defined, factor influencing metabolic outcomes after COVID-19, thereby necessitating further research with standardized follow-up intervals.

Furthermore, corticosteroids were widely administered to patients in this cohort, both prior to and during the COVID-19 illness, and are well-recognized for causing transient hyperglycemia and dyslipidemia. Bhat et al. [36] established that dexamethasone, the primary corticosteroid utilized in moderate-to-severe COVID-19, is linked to exacerbated hyperglycemia in individuals with diabetes due to mechanisms such as increased insulin resistance and hepatic gluconeogenesis, and it did not significantly enhance survival rates in this demographic. This indicates that the metabolic risks associated with dexamethasone should be carefully considered in patients with pre-existing T2D.

This study has various limitations that must be addressed when evaluating the results. First, the retrospective, observational design does not allow for causal inference. In particular, the lack of a contemporaneous non-infected T2D control group means that the observed pre-to-post changes cannot be attributed specifically to COVID-19 infection, as they may also reflect the natural course of diabetes management, temporal trends, treatment changes, or broader pandemic-related factors such as altered healthcare access, lifestyle changes, or lockdown-related behavior.

Second, 304 patients were excluded because of missing data, and relying on existing health records increases the risk of poor or inconsistent documentation of comorbidities, medication changes, and laboratory values. Third, the interval between COVID-19 infection and post-infection laboratory testing varied among patients and was significantly associated with triglyceride levels, indicating that heterogeneous follow-up timing may have influenced the observed glycemic and lipid outcomes; standardized follow-up intervals were not feasible given the retrospective design. Fourth, while medication use was recorded before, during, and after infection, changes in dosing, timing, or adherence to glucose-lowering, lipid-lowering, and corticosteroid therapies could not be fully adjusted for, leaving residual confounding as a possible explanation for the modest changes in lipid parameters. Furthermore, information on COVID-19 disease severity, hospitalization status, vaccination status, and infecting viral variant was unavailable. The absence of this data limits interpretation because these factors may influence the magnitude and direction of metabolic changes following COVID-19 infection. Data on oxygen therapy and other forms of respiratory support were also unavailable. Although corticosteroid use was documented, the available data did not permit its use as a reliable proxy for COVID-19 severity because it could not be consistently determined whether corticosteroids were prescribed specifically for COVID-19 or for other clinical indications. Consequently, severity-stratified analyses could not be performed. The findings should therefore be interpreted as descriptive pre-to-post changes within this cohort and should not be generalized as uniform metabolic effects of COVID-19. The multivariable regression models accounted for only a small fraction of the variance in lipid outcomes, indicating that unmeasured variables such as dietary habits, physical activity levels, and fluctuations in body weight probably played a role in the observed relationships. In addition, some comorbidities included in the exploratory regression models were uncommon, particularly lupus erythematosus, which was present in only four patients. Consequently, coefficient estimates for these rare conditions may be unstable and should be interpreted cautiously. Finally, while several of the observed differences were statistically significant, their magnitudes were small, and the clinical relevance of these changes—particularly for long-term cardiovascular risk in this population—needs to be confirmed in prospective studies with longer follow-up and matched controls.

## 5. Conclusions

This study revealed that modest changes in lipid parameters, but not glycemic markers, were observed in T2D patients following COVID-19 infection. These findings highlight the importance of monitoring lipid profiles after COVID-19 infection in patients with T2D. Future prospective controlled studies are needed to determine whether the observed lipid changes are causally related to SARS-CoV-2 infection and to better define their long-term clinical significance.

## Figures and Tables

**Table 1:** Demographic and clinical characteristics of the study cohort.

Variables	Descriptive Statistics (*N*/%)
Total number of patients	969
Sex (female), *n* (%)	549 (56.7)
Age (y), median (IQR)	58 (49–67)
Pre-COVID interval, days, median (IQR)	126 (74–203)
Post-COVID interval, days, median (IQR)	129 (79–211)
Hypertension, *n* (%)	499 (51.5)
Coronary artery disease, *n* (%)	14 (1.4)
COPD, *n* (%)	9 (0.9)
Asthma, *n* (%)	138 (14.2)
ESRD, *n* (%)	32 (3.3)
CKD, *n* (%)	70 (7.2)
Osteoporosis, *n* (%)	22 (2.3)
Rheumatoid arthritis, *n* (%)	10 (1.0)
Lupus erythematosus, *n* (%)	4 (0.4)

Data are presented as numbers (percentages) unless otherwise indicated. Abbreviations: CKD, chronic kidney disease; COPD, chronic obstructive pulmonary disease; ESRD, end-stage renal disease; IQR, interquartile range.

**Table 2:** Medication classes and percentage of use among patients before, during, and after COVID-19 infection.

Therapeutic Class	Medication	Percentage of Patients Using the Medication (%)
**Systemic Corticosteroids**	Betamethasone	26.28
	Dexamethasone	48.33
	Hydrocortisone	45.32
	Methylprednisolone	22.38
	Prednisone	20.38
**Inhaled/Intranasal Corticosteroids**	Budesonide	35.63
	Fluticasone	6.68
	Mometasone	47.33
	Triamcinolone	6.24
**Antidiabetic Agent**	Metformin	90.09
**Lipid-Lowering Agent**	Statins	95.55

**Table 3:** Paired changes in lipid parameters following COVID-19 infection.

Parameter	*n*	Pre-COVID-19	Post-COVID-19	HL Change *	Effect Size ^†^	*p*-Value ^‡^
TG	686	1.47 (1.13–2.01)	1.44 (1.08–1.94)	−0.040 (−0.080 to 0.001)	−0.090	0.042
TC	740	4.26 (3.67–5.03)	4.14 (3.55–4.93)	−0.070 (−0.125 to −0.020)	−0.112	0.008
LDL-C	741	2.69 (2.11–3.45)	2.53 (1.98–3.23)	−0.125 (−0.175 to −0.080)	−0.217	<0.001
HDL-C	730	1.04 (0.90–1.21)	1.04 (0.89–1.22)	−0.005 (−0.015 to 0.010)	−0.021	0.632

All concentrations and changes are expressed in mmol/L. * HL change was calculated as post-COVID-19 minus pre-COVID-19; negative values indicate reductions. ^†^ Effect size is the matched-pairs rank-biserial correlation. ^‡^ *p*-values were calculated using the two-tailed Wilcoxon matched-pairs signed-rank test. **Abbreviations:** COVID-19, coronavirus disease 2019; HDL-C, high-density lipoprotein cholesterol; HL, Hodges–Lehmann; IQR, interquartile range; LDL-C, low-density lipoprotein cholesterol; TC, total cholesterol; TG, triglycerides.

**Table 4:** Univariate correlation between triglycerides, total cholesterol, and low-density lipoprotein (LDL) and baseline characteristic variables.

Independent Variables	Dependent Variables					
	Triglycerides		Total Cholesterol		Low-Density Lipoprotein (LDL)	
	Correlation Coefficient	*p*-Value	Correlation Coefficient	*p*-Value	Correlation Coefficient	*p*-Value
Sex	0.094	0.014 *	−0.022	0.554	0.031	0.396 *
Age	0.016	0.674	−0.011	0.764	0.001	0.975
Hypertension	0.025	0.511	0.018	0.616	0.037	0.314 *
Coronary artery disease	0.012	0.748	0.048	0.216 *	−0.076	0.037 *
COPD	−0.035	0.360 *	−0.044	0.230 *	−0.019	0.602
Asthma	0.002	0.956	−0.015	0.681	−0.085	0.021 *
ESRD	0.051	0.184 *	−0.038	0.300 *	−0.076	0.039 *
CKD	0.029	0.444 *	−0.032	0.391 *	−0.069	0.060 *
Osteoporosis	−0.015	0.694	0.003	0.931	0.026	0.0483 *
Rheumatoid arthritis	−0.035	0.358 *	0.006	0.861	−0.025	0.489 *
Lupus erythematosus	−0.066	0.083 *	−0.013	0.720	0.021	0.562
Time between pre-COVID-19 test and COVID-19 infection	−0.025	0.514	0.018	0.618	−0.001	0.972
Time between COVID-19 infection and post-COVID-19 test	0.072	0.059 *	0.046	0.214 *	0.035	0.339 *

COPD, Chronic Obstructive Pulmonary Disease; ESRD, End-Stage Renal Disease; CKD, Chronic Kidney Disease. * denotes a *p*-value below 0.5, representing variables then included in the multivariate regression model.

**Table 5:** Multivariate regression model for triglycerides, total cholesterol, and low-density lipoprotein.

Dependent Variables								
Triglyceride			Total Cholesterol			Low-Density Lipoprotein (LDL)		
Independent Variables	Coefficients (B)	R Square	Independent Variables	Coefficients (B)	R Square	Independent Variables	Coefficients (B)	R Square
Sex	0.095 **	0.159	Coronary artery disease	0.024	0.007	Sex	0.028	0.016
COPD	−0.008		COPD	−0.044		Hypertension	0.020	
ESRD	0.031		ESRD	−0.018		Coronary artery disease	−0.057	
CKD	0.026		CKD	−0.032		Asthma	−0.061	
Rheumatoid arthritis	−0.025		Time between COVID-19 infection and post-COVID-19 test	0.042		ESRD	−0.023	
Lupus erythematosus	−0.123 ***					CKD	−0.048	
Time between COVID-19 infection and post-COVID-19 test	0.365 ***					Osteoporosis	0.029	
						Rheumatoid arthritis	−0.045	
						Time between COVID-19 infection and post-COVID-19 test	0.023	

COPD, Chronic Obstructive Pulmonary Disease; ESRD, End-Stage Renal Disease; CKD, Chronic Kidney Disease. ** and *** denote a significant association (** *p* < 0.01, *** *p* < 0.001).

## Data Availability

Might be provided upon request to the corresponding author.

## Supplementary Materials

The following supporting information can be downloaded at: https://www.mdpi.com/article/10.3390/pathogens15090934/s1, Table S1: Characteristics of the outcome-specific paired analytical samples; Table S2: Univariate correlation between triglyceride, total cholesterol, and low density lipoprotein (LDL) and variables of baseline characteristics; Table S3: Triglyceride changes by post-COVID-19 testing interval.
