# Supplementary material for: Changes in Glycemic Control and Lipid Profiles Following COVID-19 Infection in Adults with Type 2 Diabetes: A Retrospective Study in Saudi Arabia
Source: Pathogens. 2026 Sep 4;15(9):934. doi: 10.3390/pathogens15090934 (PMC13610501; doi:10.3390/pathogens15090934)
Supplement: Supplementary file 1 [file pathogens-15-00934-s001.zip › pathogens-4521930-supplementary.pdf]

Supplemental Table S1. Characteristics of the outcome-specific paired analytical samples

**Characteristic**	**FBG**	**HbA1c**	**TG**	**TC**	**LDL-C**	**HDL-C**
Paired observations, n	764	686	686	740	741	684
Female, n (%)	439 (57.5)	396 (57.7)	381 (55.5)	418 (56.5)	418 (56.4)	390 (57.0)
Age, years, median (IQR)	57 (48–65)	58 (50–66)	58 (49–66)	58 (49–66)	58 (49–66)	58 (49–66)
Hypertension, n (%)	399 (52.2)	356 (51.9)	359 (52.3)	385 (52.0)	386 (52.1)	381 (55.7)
Coronary artery disease, n (%)	8 (1.0)	11 (1.6)	11 (1.6)	13 (1.8)	13 (1.8)	13 (1.9)
COPD, n (%)	3 (0.4)	6 (0.9)	2 (0.3)	5 (0.7)	5 (0.7)	5 (0.7)
Asthma, n (%)	97 (12.7)	104 (15.2)	92 (13.4)	102 (13.8)	103 (13.9)	99 (14.5)
ESRD, n (%)	22 (2.9)	22 (3.2)	18 (2.6)	22 (3.0)	20 (2.7)	20 (2.9)
CKD, n (%)	47 (6.2)	43 (6.3)	43 (6.3)	46 (6.2)	47 (6.3)	47 (6.9)
Osteoporosis, n (%)	13 (1.7)	12 (1.7)	12 (1.7)	14 (1.9)	14 (1.9)	14 (2.0)
Rheumatoid arthritis, n (%)	9 (1.2)	8 (1.2)	9 (1.3)	10 (1.4)	10 (1.3)	10 (1.5)
Lupus erythematosus, n (%)	4 (0.5)	3 (0.4)	3 (0.4)	3 (0.4)	3 (0.4)	3 (0.4)
Pre-COVID-19 interval, days, median (IQR)	126 (74–203)	136 (79–210)	115 (68–183)	111 (67–174)	112 (68–175)	114 (68–175)
Post-COVID-19 interval, days, median (IQR)	129 (79–211)	188 (134–239)	124 (78–202)	120 (74–193)	122 (78–198)	125 (78–206)

Supplemental Table S2. Univariate correlation between triglyceride, total cholesterol, and low density lipoprotein (LDL) and variables of baseline characteristics

**Independent Variables**	**Dependent Variables**					
	**Triglyceride**		**Total Cholesterol**		**Low Density Lipoprotein (LDL)**	
	correlation coefficient	*p value*	correlation coefficient	*p value*	correlation coefficient	*p value*
Sex	0.094	0.014*	-0.022	0.554	0.031	0.396*
Age	0.016	0.674	-0.011	0.764	0.001	0.975
Hypertension	0.025	0.511	0.018	0.616	0.037	0.314*
Coronary artery disease	0.012	0.748	0.048	0.216*	-0.076	0.037*
COPD	-0.035	0.360*	-0.044	0.230*	-0.019	0.602
Asthma	0.002	0.956	-0.015	0.681	-0.085	0.021*
ESRD	0.051	0.184*	-0.038	0.300*	-0.076	0.039*
CKD	0.029	0.444*	-0.032	0.391*	-0.069	0.060*
Osteoporosis	-0.015	0.694	0.003	0.931	0.026	0.0483*
Rheumatoid Arthritis	-0.035	0.358*	0.006	0.861	-0.025	0.489*
Lupus erythematosus	-0.066	0.083*	-0.013	0.720	0.021	0.562
Time between pre-COVID19 test and COVID19 infection	-0.025	0.514	0.018	0.618	-0.001	0.972
Time between COVID19 infection and post-COVID19 test	0.072	0.059*	0.046	0.214*	0.035	0.339*

COPD; Chronic Obstructive Pulmonary Disease, ESRD; End-Stage Renal Disease, CKD; Chronic Kidney Disease.* *p value* below 0.5 which then included in the multivariate regression model

Supplementary Table S3. Triglyceride changes by post-COVID-19 testing interval:

Post-COVID-19 interval	n (%)	Pre-COVID-19	Post-COVID-19	Δ TG	*p*-value
1–3 months (30–90 days)	219 (31.9)	1.43 (1.10–2.11)	1.47 (1.08–1.92)	−0.07 (−0.36 to 0.22)	0.065
>3–6 months (91–180 days)	265 (38.6)	1.55 (1.21–2.07)	1.51 (1.10–2.07)	−0.02 (−0.29 to 0.21)	0.370
>6–12 months (181–365 days)	202 (29.4)	1.37 (1.10–1.91)	1.37 (1.07–1.80)	−0.02 (−0.35 to 0.32)	0.486

Data are presented as median (IQR), unless otherwise indicated. TG concentrations and ΔTG are expressed in mmol/L. ΔTG was calculated as post-COVID-19 TG minus pre-COVID-19 TG; negative values indicate a reduction. Within-group pre–post comparisons were conducted using the Wilcoxon signed-rank test.

Abbreviations: COVID-19, coronavirus disease 2019; TG, triglycerides; ΔTG, change in triglyceride concentration; IQR, interquartile range.
